# Marine cleaning stations as hotspots for cryptobenthic reef fish

**DOI:** 10.1038/s41598-026-44074-7

**Published:** 2026-04-02

**Authors:** C. G. Obst, P. Vetter, R. L. Gunn

**Affiliations:** Animal Evolutionary Ecology, Institute of Evolution and Ecology, University of Tübingen, 72076 Tübingen, Germany

**Keywords:** Cleaning mutualisms, Obligate cleaner species, Cryptobenthic reef fish, Fish diversity, Symbiotic relationships, Third party species, Ecology, Ecology, Ocean sciences

## Abstract

Species interactions are fundamental drivers of biodiversity and often have benefits that extend beyond the directly interacting parties. Within coral reef ecosystems, cleaning mutualisms whereby ‘cleaner’ species remove ectoparasites from ‘client’ species at dedicated cleaning ‘stations’ are well documented, yet the indirect benefits remain relatively unknown. Specifically, cleaning stations may act as a refuge via predation mitigation, which could indirectly benefit functionally important reef fishes, such as cryptobenthic reef fishes (CRF). CRF are the most abundant group of reef fishes, display rapid turnover due to high predation and are vital to marine food webs. We provide novel evidence of an association between CRF communities and the presence of obligate cleaner goby (*Elacatinus lobeli*) cleaning stations in the Caribbean. Through in situ*,* visual surveys, we found a significantly higher abundance and diversity of CRF within the benthos around *E. lobeli* cleaning station coral heads, compared to the benthos outside of cleaning stations and around coral heads with no cleaners present. We highlight the importance of the indirect benefits of species interactions on third party species. Marine cleaning stations may help maintain functionally important fish communities, with cascading consequences for coral reef food webs and ecosystem functioning.

**Supplementary Information:**

The online version contains supplementary material available at 10.1038/s41598-026-44074-7.

## Introduction

The interconnectedness of Species on Earth results in complex interactions that significantly influence the structure and function of ecosystems. Such interactions can be classified as positive or negative. Negative species interactions include predation, whereby a predator consumes a prey organism, with a clear benefit for the predator and a cost to the prey. Although only the predator directly benefits, predation can also have indirect effects on other species. For example, the removal of a prey species can lead to an increase in the abundance of its competitors^1^. Positive interactions between species involving the trading of goods or services lead to mutual benefits for all interacting parties^2^. These so-called mutualistic interactions are ubiquitous across ecosystems and are vital in maintaining ecosystem diversity and stability^3^. Pollination is a classic example, whereby insects and birds obtain nectar from plants whilst subsequently facilitating pollen transfer and promoting plant reproduction^4,5^. This mutualistic interaction extends beyond direct benefits, promoting biodiversity and ecosystem stability, and is a critical component of global food webs^6,7^. A key challenge in ecological research is to unravel the complexities of species interactions, and it is necessary to identify direct and indirect relationships among species. Only then can we fully interpret the consequences of disruptions such as environmental instability for species interactions and the ecosystem as a whole^8^.

In biodiversity-rich ecosystems such as coral reefs, species interactions promote niche partitioning and a remarkable diversification of life strategies that enable local coexistence^9^. Coral reefs are therefore an ideal system to study the complexities of species interactions. Corals themselves are reliant on symbioses with photosynthetic Zooxanthellae. Zooxanthellae provide energy to the coral through photosynthetic byproducts and the coral provides habitat and shelter for the Zooxanthellae^3^. Another prominent interaction within coral reef ecosystems is the cleaning mutualism. So called ‘cleaner’ species feed on and remove ectoparasites from ‘client’ species^10,11^. In the Caribbean, cleaner gobies (*Elacatinus spp.*) and cleaner shrimp (*Ancylomenes pedersoni*) serve as dedicated cleaners, deriving most of their nutritional resources via cleaning at specific cleaning ‘stations’. These cleaning stations consist of structurally complex live boulder coral for the cleaner gobies, and the corkscrew anemone (*Bartholomea annulata*) for the cleaner shrimp^10,12,13^.

The basic functioning of marine cleaning mutualisms and the consequences for directly interacting parties are generally well-understood. Less attention has been given to the implications of these mutualisms for third party species, i.e. species indirectly associated with a species interaction. For example, healthy coral profiting from the coral-Zooxanthellae-symbiosis provides habitat, refuges, and breeding grounds for coral reef fishes^3^. In cleaning mutualisms, cleaning interactions can influence habitat selection for juvenile fish^14,15^, and cleaning stations provide microhabitats that support a diverse range of third-party species^16^. The presence of cleaner fish has also been associated with reduced aggressive behaviour in client fish^17^. This is likely due to reduced stress levels in client fish due to tactile stimulation of clients by cleaners^18^. Cleaner species could therefore play a significant role in mitigating predator aggression toward bystander fish. In turn, cleaner presence could provide third party species such as juvenile damselfish *Pomacentrus amboinensis*^14,15^, with a refuge from predation^17^. However, the indirect influence of cleaning stations on fish communities remains understudied, and existing research has focussed on a limited scope of reef fish communities^14,15,19,20^. This gap may stem from inadequate methods for assessing biodiversity among diverse fish groups with varying body sizes, behaviours and ecologies^21^.

**Fig. 1:**
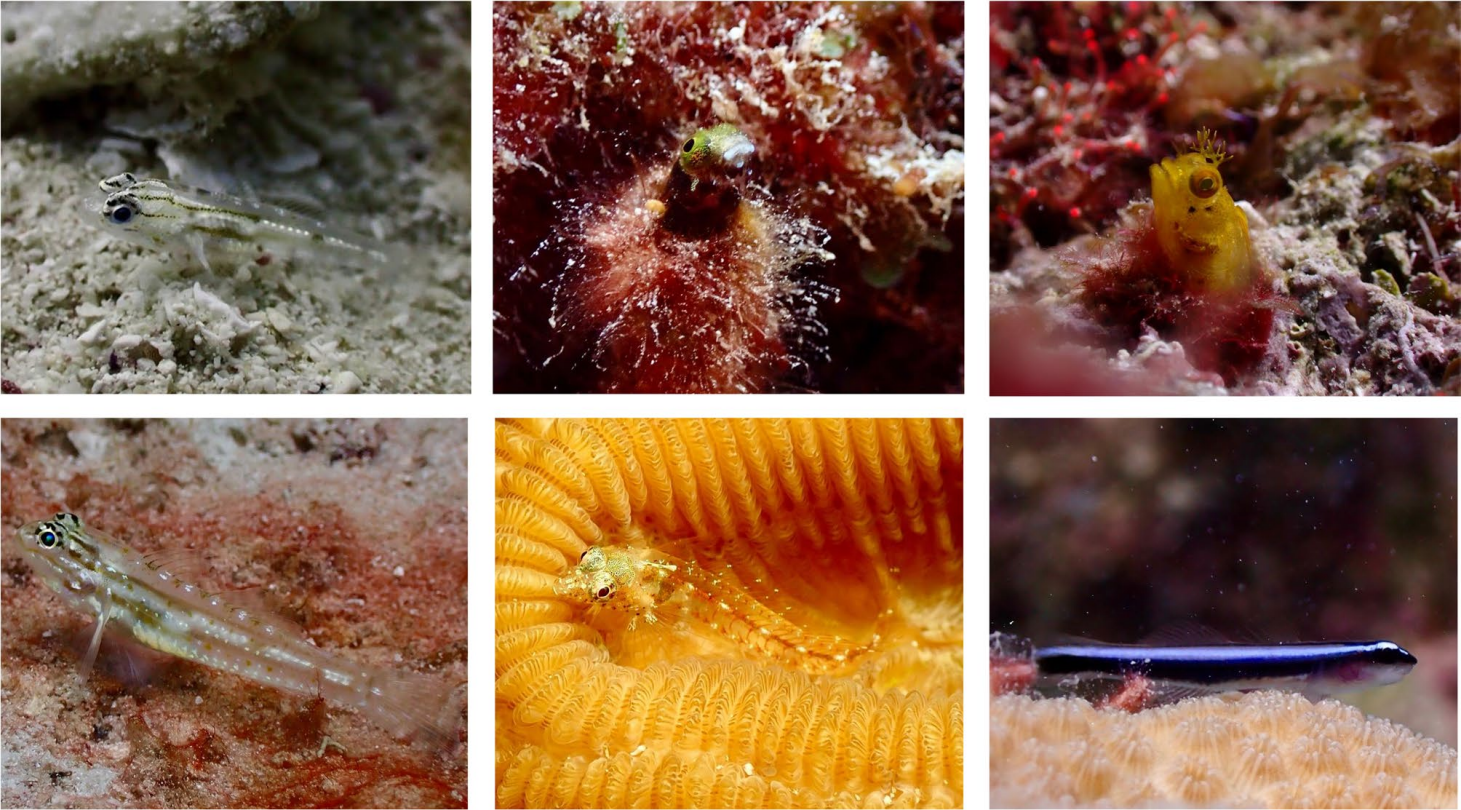
Cryptobenthic reef fishes: Top, left to right: bridled goby (*Coryphopterus glaucofrauneum*), spinyhead blenny (*Acanthemblemaria spinosa*), female roughhead blenny (*Acanthemblemaria aspera*). Bottom, left to right: bartail goby (*Coryphopterus thrix*), female western smoothhead glass blenny (*Emblemariopsis pricei*), Caribbean neon goby (*Elacatinus lobeli*). Images: Chiara Obst.

One group that has been largely neglected in cleaning mutualism research is cryptobenthic reef fishes (hereafter referred to as CRF). CRF are typically small (< 5 cm), visually or behaviourally cryptic and reside near or within the benthos^22^. CRF comprise a phylogenetically diverse set of fish families (Fig. 1). They contribute significantly to ecosystem functioning, accounting for ~ 50% of the fish abundance on a reef. CRF therefore represent the most individual-rich group of vertebrates within coral reef ecosystems^23,24^. As many CRF species do not live beyond two years, turnover rates are exceptionally high^25^. They play a vital role in the marine food web by serving as a food source, offering readily available and quickly replenished fish tissue for consumption^26^. This exposes CRF to a significant predation pressure and evolutionarily favours traits that reduce this pressure^12,27^. Contrary to conventional understanding, a study by Harborne et al.^28^, found a positive association between dead coral cover and CRF abundance and diversity. Live coral may harbour more predators and given CRF’s high susceptibility to predation, rubble could prove to be a more advantageous habitat^29^. However, as cleaning stations have been associated with reduced predation and aggression^17,30^, cleaning station live coral and associated habitat could provide a refuge for CRF.

CRF often consume detritus, to varying extents, with a diet containing a variety of microscopic prey items often inaccessible for larger species^24,31,32^. During the process of cleaning, particles may fall to the ground and increase the abundance of detritus around cleaning stations. Therefore, cleaning stations might additionally provide a nutritional benefit for the neighbouring cryptobenthic fish.

To elucidate the ecological relationships within the Caribbean cleaner Goby mutualism and how the cleaning system shapes marine networks on a local scale, we aimed to investigate the relationship between Caribbean Goby cleaning stations and CRF communities. We provide an overall comparison on the use of two methods to quantify CRF abundance and diversity around cleaning stations and control areas of the reef: underwater visual census (UVC) and the application of clove oil. We surveyed CRF using quadrats (0.5 m^2^) (n = 5 per replicate) placed on the benthos around 21 active cleaning stations and 21 benthic areas with no cleaning stations (‘control’ areas). Surveys consisted of a 3-min UVC survey followed by the application of clove oil. We additionally conducted 3-min visual searches of similarly sized live star coral heads: 14 coral heads that were active cleaning stations, and 14 that did not have cleaners present at the time of surveys and were therefore not active cleaning stations.

We also conducted Habitat assessment scores (HAS) to control for variation in habitat structure among replicates. We hypothesized that the abundance, species richness, alpha diversity, and community structure of CRF would be greater on (1) the benthos around cleaning stations compared to control areas, and (2) on active cleaning station coral heads compared to coral heads with no cleaner present. Associations between cleaning stations and cryptobenthic fish communities could enhance our understanding of the persistence of a functionally important yet understudied group of reef fishes. In turn, this will highlight the importance of considering the cascading effects of species interactions for third party species.

## Results

### Does CRF abundance and diversity vary with survey method?

**Fig. 2:**
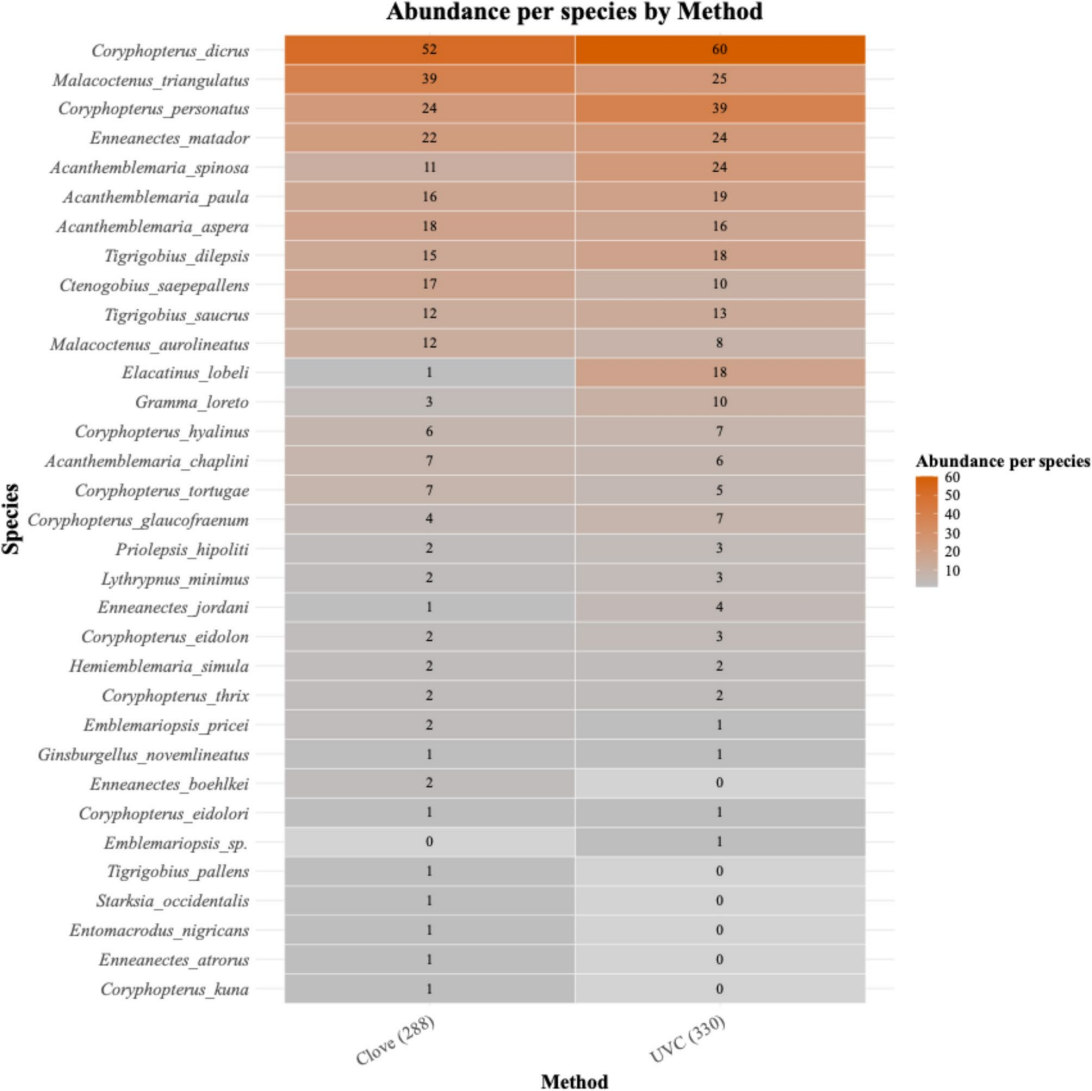
Heatmap of species found per method. Colour gradient indicates the total abundance of each species summed across 42 replicates for each method, ranging from 0 (light grey) to 60 (dark red). Numerical values next to X axis labels indicate the total fish abundance for each method. No standardization or scaling was applied.

Through the UVC benthos surveys, we identified 174 cryptobenthic fishes from 25 species around active cleaning stations and 95 cryptobenthic fishes from 21 species at the control areas (Fig. 2). All species were assigned to one of 6 diet categories: Benthivore, Detritivore, Omnivore, Carnivore, Planktivore, Ectoparasites. Of all the detrivorous individuals surveyed, 76% were found around cleaning stations (Supplementary Figure S1). In total, we found 25 CRF species using benthic UVC.

With the addition of clove oil on the benthos, we found 193 fishes from 30 species around active cleaning stations and 96 fishes from 23 species around control areas. In total we found 32 species using clove oil. There was no significant difference between the two survey methods in terms of fish abundance (Paired t-test: t = 1.5019, df = 41, *p* value = 0.1408), species richness (Paired t-test: t = − 1.1841, df = 41, *p* value = 0.2432) or diversity (Paired t-test: t = − 1.1327, df = 41, *p* value = 0.2639). Since there is no significant difference between the surveying methods, and the two methods are not independent of one another, all further analyses have been conducted with the UVC dataset only.

### Do CRF diversity metrics vary between cleaning stations and control areas?

#### Benthos around versus away from cleaning stations

**Fig. 3:**
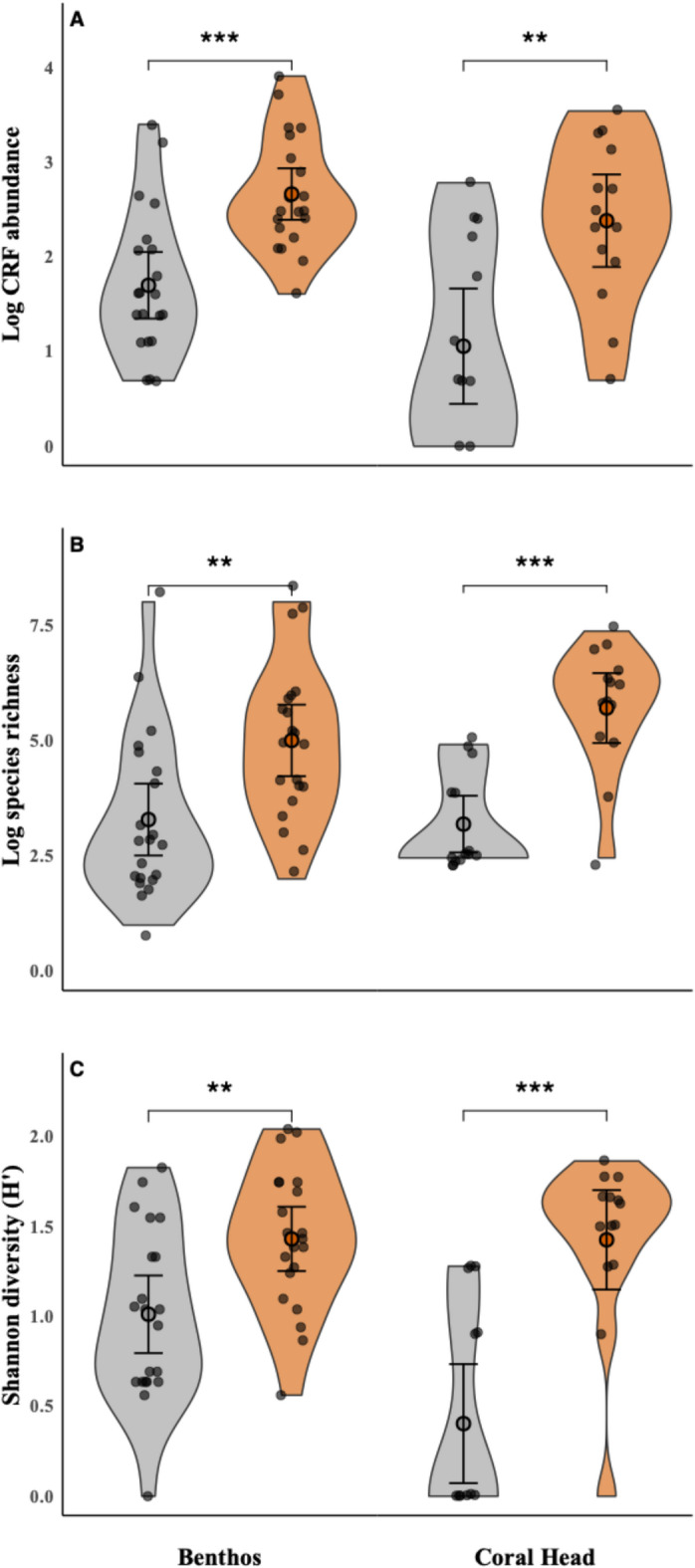
Fish community indices for the two survey types. Violin plots showing (**A**) the abundance (number of individuals per 0.5 m^2^, log-scaled), (**B**) species richness (number of species per 0.5 m^2^, log-scaled), and (**C**) diversity (H′, Shannon Diversity Index) of CRF on the benthos and on coral heads, comparing areas without cleaning stations (grey) to those with active cleaning stations (orange) (mean and 95% CI); asterisks indicate statistical significance (GLMM; alpha = 5%).

Mean (± standard deviation, SD) log-transformed CRF abundance was higher at active cleaning stations (2.67 ± 0.60) than at non-cleaning stations (1.70 ± 0.77). Similarly, CRF diversity was greater at active cleaning stations (1.43 ± 0.39) compared to non-cleaning stations (1.01 ± 0.47). Log-transformed CRF richness also increased at active cleaning stations (1.55 ± 0.36) relative to non-cleaning stations (1.07 ± 0.50; mean ± SD). The abundance (GLMM: z-value = 4.250, df = 36, *p* value < 0.001, Fig. 3A), species richness (GLMM: z-value = 3.229, df = 36, *p* value < 0.001, Fig. 3B) and the Shannon diversity index (GLMM: z-value = 2.579, df = 36, *p* value = 0.01, Fig. 3C) of CRF were significantly higher around the benthos of active cleaning stations (CS) than around benthic areas without a cleaning station (no CS) (Fig. 3). The model also indicated a significant effect of the HAS on fish abundance (GLMM: z-value = 2.467, df = 36, *p* value = 0.0136), species richness (GLMM: z-value = 3.502, df = 36, *p* value < 0.001) and diversity (GLMM: z-value = 3.851, df = 36, *p* value < 0.001). For all models, the random effects of Station and Depth explained a maximum of 20% of variation in the model (max Station: 0.198; max Depth: < 0.01) (Supplementary Table S1). The interaction term of the linear model between HAS and Station status (active CS/no CS) shows no significance for abundance (LM Estimate: − 0.2034, (0.2076 SE), *p* value = 0.334), species richness (LM Estimate: 0.001432, (0.124349), *p* value = 0.991) and diversity (LM Estimate: 0.05447, (0.12154), *p* value = 0.6566).

#### Coral heads with versus without cleaners’ present

Through the UVC coral head surveys, we identified 61 fishes from 18 species on coral heads with a cleaner present and 22 fishes from 10 species on coral heads without a cleaner. To directly compare CRF on cleaning station habitat with and without the presence of *E. lobeli* cleaners, and to address the problem of differences in habitat composition between the benthos around active cleaning stations and benthic areas without a cleaning station, we additionally surveyed CRF on live coral heads. Mean (± SD) log-transformed coral abundance was higher at coral heads where cleaners were present (2.37 ± 0.84,) than at coral heads with no cleaners present (1.05 ± 1.05). Similarly, log-transformed coral species richness was greater at coral heads with cleaners present (1.61 ± 0.37) relative to coral heads without cleaners (0.90 ± 0.30), and Shannon diversity was also higher at coral heads with cleaners present (0.83 ± 0.28) compared to coral heads without cleaners (0.23 ± 0.33). Similarly to the analyses above, the abundance (GLMM: z-value = 3.621, df = 21, *p* value < 0.001, Fig. 3A; HAS: *p* value = 0.515), species richness (GLMM: z-value = 5.772, df = 21, *p* value < 0.00, Fig. 3B; HAS: *p* value = 0.567) and diversity (GLMM: z-value = 4.446, df = 21, *p* value < 0.001, Fig. 3C; HAS: *p* value = 0.858) were higher on coral heads where cleaners were present, i.e. active cleaning stations compared to coral heads with no cleaners present (Fig. 3). Importantly, habitat assessment scores had no significant effect for abundance (LM Estimate: 1.1047, (0.5499 SE), *p* value = 0.0570), species richness (LM Estimate: 0.3770, (0.2071), *p* value = 0.08234) and diversity (LM Estimate: 0.2824, (0.1912), *p* value = 0.154).

### Does CRF community composition vary between cleaning stations and control areas?

**Fig. 4:**
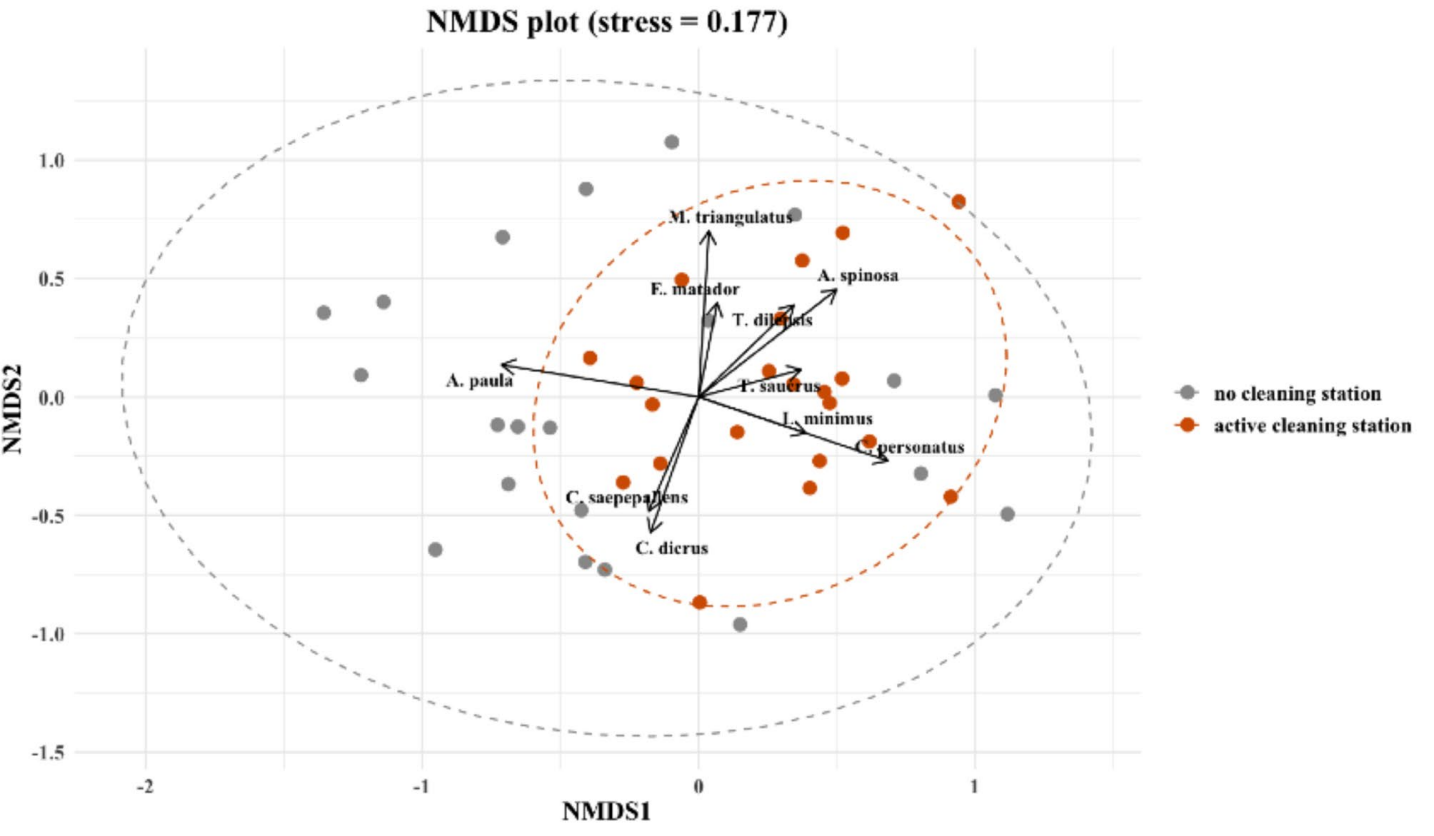
Non-metric multidimensional scaling (NMDS) for CRF community visualisation between the benthos around active cleaning stations and the benthos where no cleaning station was present. NMDS axes are dimensionless and represent relative differences in community structure based on rank dissimilarities. Distances among points indicate similarity in species composition. Species vectors (arrows) indicate taxa significantly associated with the ordination; arrow length reflects the strength of the association and direction indicates increasing abundance. (Stress = 0.1772768).

The cryptobenthic fish community composition differed significantly between the benthos around cleaning stations and the benthos with no cleaning station present, with about 6.6% of the variation explained by the presence of an active cleaning station (PERMANOVA: F_1,40_ = 2.99, *p* value = 0.003). These results were ranked by an ANOSIM with an (R) statistic of 0.1388 indicating a low level of dissimilarities in community composition between the station statuses (CS/no CS) and indicating that the small differences in community composition at the benthos around cleaning stations are statistically significant, with a low probability that these differences could have arisen by chance. A multilevel pattern analysis of the ANOSIM results demonstrated significant species associations with station status (Fig. 4). Significant associations with active cleaning stations included the Matador Triplefin (*Ennaeanectes matador*) (*p* value = 0.017), Colon Goby (*Coryphopterus dicrus*) (*p* value = 0.009) and the Saddled Blenny (*Malacoctenus triangulates*) (*p* value = 0.045). For the control habitat sections without a cleaning station only the Dwarf Spinyhead Blenny (*Acanthemblemaria paula*) (*p* value = 0.0052) showed a significant association. The NMDS produced a moderate stress level of 0.177, thus the visual representation of the dissimilarity between the communities should be treated with caution (Fig. 4).

## Discussion

Marine cleaning mutualisms can trigger a host of indirect interactions for coral reef communities^20,33^. We provide what could be the first preliminary link between the presence of marine cleaning stations and the abundance and diversity of CRF. As CRF play a fundamental role in the marine food web, understanding potential habitat preferences of these fishes can scale up to have implications for coral reef ecosystems at higher ecological levels.

We show that the abundance, species richness, and diversity of cryptobenthic fish was higher on the benthos around cleaning stations and on active cleaning station coral heads compared to control areas (benthos and coral heads) with no cleaners present. Our results conform to work on other third-party species associated with cleaning stations, such as juvenile Ambon damselfish (*Pomacentrus amboinensis)*^14,15^). In this case, Sun et al.^14^ suggested that cleaner presence could indicate microhabitat quality to juvenile fishes, due to the presence of conspecifics or other sensory cues. Cleaning stations are more structural complex with a higher refuge availability than non-cleaning station coral heads^13^. This suggests that cleaner fish may also use microhabitat quality as a settlement cue. Additionally, juveniles are particularly vulnerable to predation^14,15^, and there is evidence of cleaning stations acting as refuges against predation^17^. Access to a refuge with less predatory aggression is particularly beneficial for the developmental stages of reef fishes^14,15^.

Similarly, we offer three possible benefits for CRF settling around active cleaning stations. Firstly, CRF are highly vulnerable to predation throughout their life history^25^. As a result, actively settling near cleaning stations may provide additional refuge advantages to their typically preferred habitats such as rubble^28,34,35^. Secondly, CRF may benefit from cleaning services themselves. Benthic-associated fishes may be more susceptible to parasite infection than pelagic species^36^. Juvenile fishes of similar size to CRFs benefit from being cleaned at cleaning stations^15^. Furthermore, Grutter. (2010)^11^ found that the cleaner wrasse *Labroides dimidiatus* is cleaned by its own conspecifics. Whilst undocumented to our knowledge, it is possible that something similar is true between CRF species. Finally, CRF often utilize detritus as a food source^32,37^. Cleaning stations could therefore provide CRF with better access to nutritional resources, as particles (e.g. skin, scales, parasites) may fall to the benthos during the cleaning process^38^. Even though it is still difficult to distinguish between the fine scaled differences in diets of CRF, Brandl et al.^31^ suggests that all CRF might feed on microscopic prey and detritus. In our work, 76% of all the CRF with a primarily detrivorous diet were found around active cleaning stations. CRF could therefore benefit from additional refuges, health and nutritional resources from settling around active cleaning stations.

The community structure of CRF varied between communities on the benthos around cleaning stations and benthic areas without a cleaning station. Fish that showed a significant association with the presence of cleaning stations were substrate-dwelling species, rather than tube- or free-living species. Substrate dwelling species are more visible to predators from above, resulting in a higher susceptibility to predation^34^. In contrast, tube-living blennies were the only fishes that were significantly associated with a specific habitat than with the presence of cleaning stations. These species inhabit tubes previously occupied by polychaete species, and the tubes offer protection from predation^39^. Therefore, settling near cleaning stations might be more favourable for predator-prone cryptobenthic species than for those with established predator avoidance strategies.

Studies have shown that cryptobenthic fish are underestimated in biodiversity monitoring surveys using underwater visual census^21,40–42^. We therefore included the additional method clove oil to maximise sampling effort. Surprisingly, there was no significant difference in the abundance and diversity of CRF between the two sampling methods. As fish are inevitably disturbed via the use of clove oil, the visual census was always carried out prior to the application of clove oil, causing unavoidable bias. There was also taxonomic variation in the response to clove oil^43^, with gobies (Gobiidae) affected even at low doses, whilst Triplefin (Tripterygiidae) were largely unaffected. The lack of difference between the two study methods could be a consequence of methodological bias, which was inherently difficult to control for in our field methods. Our inclusion of just the UVC data in the statistical analyses aimed to reduce the impact of such bias on our results. Nonetheless, these caveats further highlight the difficulties of sampling certain groups of reef fishes, such as CRF, which may drive the underrepresentation of such groups in ecological research.

It is commonly accepted that more complex habitats support a greater variety species^44^. Our statistical models indicated a positive effect of habitat on CRF metrics, but associations between CRF diversity metrics and cleaning station presence remained significant after accounting for habitat variation. As stated above, there is evidence that cleaners select cleaning stations based on habitat quality^13^, and CRF may do the same^28^. The higher diversity of CRF around cleaning stations could therefore be a consequence of habitat quality rather than an effect of the cleaning mutualism itself. However, our study is purely observation. We cannot make causal statements about whether CRF actively settle at cleaning stations or if *Elacatinus* cleaners actively settle in areas of high CRF abundance and diversity. Subsequently, we cannot explicitly decouple the role of habitat and cleaning station in driving CRF settlement and subsequent abundance and diversity. Experimental research removing cleaner fish and monitoring the changes in CRF communities, or experiments into the extent of a refuge effect against predators, would provide more causal evidence for the link between cleaners and CRF.

Cryptobenthic fishes play a fundamental role in the trophic cascade as they are a major food source that drives reef fish biodiversity^25^. Through an observational study, we have shown that *Elacatinus* cleaning stations could act as a biodiversity hotspot for CRF. This novel finding is the first step in answering additional questions about the ecology, behaviour, structure and function of a keystone group of reef fishes and the role of mutualistic interactions in promoting the diversity and maintenance of coral reef ecosystems.

## Methods

### Study site and station selection

This in situ observational study was conducted on a fringing reef off the Island of Utila, Islas de Bahia, Honduras (16° 05′ 17.9″ N 86° 54′ 41.0″ W) and all observations took place between the 26th of June 2024 until the 29th of July 2024. Data was collected via SCUBA, with two 50-min dives conducted per day between 11am and 3 pm to maximum depth of 6 m. We completed the following surveys for this study: Benthic surveys using quadrats (n = 5 per replicate) and both UVC and clove oil methods (n = 42), and coral head only surveys (n = 28) using a single 3 min visual search per coral head. We sampled the benthos around 21 active cleaning stations and 21 control areas with no cleaning station present. Both the benthos around active stations and areas without a cleaning station were randomly distributed across the reef, pending their availability. Benthic areas around active cleaning stations were defined by Coral heads with at least one cleaner goby (*Elacatinus lobeli)* present. *E. lobeli* is a dedicated cleaner that uses bulky coral heads as cleaning stations^45^. Areas of benthos without a cleaning station were defined as areas without any cleaner fish present or bulky coral head nearby that could potentially be occupied by a cleaner. These definitions were chosen to exclude the possibility of counting coral heads as (1) non-active stations where the cleaner fish was not visible during a visit but generally present or (2) had just abandoned the station shortly before sampling. Due to the proximity of coral heads on the sampling reef, communities around the coral heads could not have been assigned to one specific coral head. Therefore, control areas with a large distance (min. 15 m) to coral heads as active or potential cleaning stations were chosen.

We also selected an additional 28 coral heads where only the coral head itself was surveyed and not the surrounding benthos. As with the benthos surveys, the coral heads were randomly distributed across the reef. We also visually ensured that all coral heads were of approximately similar sizes and structure to reduce the amount of variation in habitat structure and composition between replicates. We surveyed 14 coral heads with a cleaner present and 14 without a cleaner present during the time of observation. This additional sampling was done to 1) Directly compare CRF communities with the presence/absence of cleaners on cleaning station habitat and 2) account for the differences between the benthos of the sampling stations with and without a cleaning station present.

### Habitat assessment

For all 42 areas of the benthos and the 28 coral heads, a habitat assessment score (HAS; following^44^, Supplementary Figure S3) was calculated to correct for the differences in habitat composition and structure among sampling sites. The HAS was assigned via direct measurement in the water prior to fish surveys by one observer, with the same observer completing the HAS for all surveys. We scored six different habitat categories (Rugosity, Variety of growth forms, Height, Refuge size categories, Live cover, Hard substratum) for each station and then calculated the mean number of all the categories summed together for statistical analysis (1). For each coral head a habitat assessment score (HAS, following^44^, Supplementary Table S2) was calculated to correct for any differences in habitat composition and structure among coral heads.

### Cryptobenthic reef fish surveys

CRF were surveyed in situ. Each Station was sampled using quadrats (0.5 m^2^) constructed from PVC pipes placed arranged in a circle directly around the station to ensure that each side was monitored. For each of the 42 areas of benthos, five replicate quadrats were sampled. First, we visually counted all present fish within each quadrat during a 3 min underwater visual census (UVC). Second, due to the underrepresentation of small cryptic fish in conventional visual survey methods^23^, we applied a weak mixture (1 part clove oil, 1 part seawater, 3 parts 70% Ethanol, renewed every 3 days) of clove oil over each quadrat after the UVC. The use of clove oil enabled us to survey any individuals not counted during the UVC, such as hidden individual’s utilising small holes for refuges, as hidden individuals then emerged in order to avoid the clove oil. Clove oil disperses immediately into the water column and in low doses is therefore a non-invasive alternative to anaesthetics such as ichthyocides which are lethal to the fish and potentially harmful to the user^46^. A 250 µm mesh net covering an area of 0.7 m^2^ was placed over the quadrat (following^29^) and the clove oil mixture was then sprayed underneath the mesh using 125 ml weighted spray bottles. After 30 s, the mesh was removed, and the quadrats were sampled. Fish were collected in a magnifying pot for photography purposes in the water, therefore never taken out of the water and all were replaced unharmed. All methods were approved under a research permit from the Honduran Government’s Instituto de Conservación Forestal. Quadrats were searched until no new fishes were found for 3 min.

For the coral head surveys, we exclusively used UVC methods, as clove oil is lethal to corals^43,46^. At each coral head, a single three-minute visual survey was conducted across the whole coral head, rather than the placement of five replicate quadrats as with the benthos surveys. A single search was conducted because (1) Quadrat placement around the coral heads was not always feasible given the placement and structure of the coral heads; (2) A three-minute search of the coral head was always enough to observe all the CRF individuals. In most cases, 2.5 min was sufficient but the full three minutes was always used and 3) Completing multiple replicate three-minute visual searches of the same area would likely have resulted in pseudoreplication of the CRF community.

### Fish identification

Along the definition of Brandl et al.^31^ of the 17 core families of CRF, we consider species from 11 families (*Apogonidae, Blenniidae, Chaenopsidae, Dactyloscopidae, Gobiidae, Gobiesocidae, Grammatidae, Labrisomidae, Pseudochromidae, Syngnathidae, and Tripterygiidae* that form the core composition of CRF in the Caribbean^47,48^. The most species-rich families are Gobiidae (gobies and the suborder Blennioidei (blenny-like fishes^31^. Although CRF are found worldwide^31^, their diversity and opposed threats remain largely unexplored in many regions.

Each fish species was photographed with an Olympus tg6 in an Olympus underwater housing. The underwater settings of the camera were used together with automatic settings for aperture and shutter speed and iso (isoMax = 800). White balance was set manually and photos taken in RAW and JPEG formats.

Additional fish that could not be easily visually identified were placed in a magnifying pot and photographed in situ from different angles. These photographs were used in combination with the use of the book “Caribbean Reef Life—A Field Guide for Divers”^48^ and the online database fishbase, to maximise the accuracy of the identification. Fishes were assigned to a family and species level always by the same person to reduce bias.

Fish species were also assigned to a feeding strategy using information from the Smithsonian Tropical Research Institute database and fishbase^54^. Nonetheless, these diets are not exclusive and species may also feed on items from multiple different categories, as there is little evidence on small-scale differences in feeding between closely related species^31^.

### Statistical analysis

All statistical analysis and graphical visualizations (ggplot2;^49^) were done using *R* (version 4.4.1; R core Team, 2024^55^). All figures using colour gradients were generated with palettes accessible to readers with common forms of colour vision deficiency. Statistical significance was accepted at an alpha level of *p* < 0.05 for all analysis. Restructuring of data frames and data organization was done using the package *tidyverse*^50^.

### CRF communities: Benthic surveys

To investigate differences between the two monitoring methods for the benthos in terms of abundance, species richness and diversity, all observational data was first split into the two methods used: underwater visual census (UVC) and clove oil. Paired t-tests were used to compare abundance, species richness and diversity between the methods (R package stats; R Core Team, 2024^55^). For all analyses, the average of all five quadrats per station was taken as a measure per station to avoid pseudoreplication.

#### Abundance

Abundance was defined as the total number of individuals at each station, which was derived by taking the average of all five quadrats per station. The abundance data was first log-transformed to ensure a normal distribution. Variables were graphically inspected for extreme values. To test for any difference in abundance between the active cleaning station and no cleaning station, a generalized linear model with a gaussian response distribution was fitted using the *R* package *glmmTMB*^51^. Station status (CS/no CS) and HAS were set as fixed factors, station and depth as random factors (model: UVC_log ~ StationStatus + HAS + (1 | Station) + (1 | Depth), family = “gaussian”). Depth was set as a random factor since the depth gradient of 2–6 m was very small. Model assumptions were checked visually with residual plots. A linear regression analysis was conducted to examine the relationship between the abundance and the HAS (R package stats, R Core Team, 2024^55^).

#### Species richness

Species richness was defined by the number of different species at each station, and the species richness data was first log-transformed to ensure normal distribution. Variables were graphically inspected for extreme values. To analyse if the species richness diversity between the active cleaning station and no cleaning station differed, a generalized linear model with a gaussian response distribution was fitted using the *R* package *glmmTMB*^51^. Station status (CS/no CS) and HAS were set as fixed factors, Station and Depth as random factors (model: richnessUVC_log ~ StationStatus + HAS + (1|Station) + (1 | Depth), family = “gaussian”). Model assumptions were checked visually with residual plots. A linear regression analysis was conducted to examine the relationship between the species richness and the HAS (R package stats, R Core Team, 2024^55^).

#### Diversity

Diversity was calculated using the Shannon’s diversity index, H’, whereby:

$$H^{\prime}=- \sum {p}_{i} ln ({p}_{i})$$

such that:

$${p}_{i}= \frac{n}{N}$$

In this context,${p}_{i}$ represents the proportion of *i-th* species in the CRF community*, n* is the number of individuals of a given species, and *N* indicates the total number of individuals in the CRF community.

Variables were graphically inspected for extreme values. To analyse if the diversity between the active cleaning station and no cleaning station differed, a generalized linear model with a gaussian response distribution was fitted using the *R* package *glmmTMB*^51^. Station status (CS/no CS) and HAS have been set as fixed factors, station and depth as random factors (model: DiversityUVC ~ StationStatus + HAS + (1 | Station) + (1 | Depth), family = “gaussian”). Model assumptions were checked visually with residual plots. A linear regression analysis was conducted to examine the relationship between the diversity and the HAS (R package stats, R Core Team, 2024^55^).

#### Species composition/fish community

Permutational analysis of variance (PERMANOVA) was used to analyse fish community differences among station statuses (CS/no CS) (R package vegan^52^). An analysis of similarity (ANOSIM) was conducted to statistically quantify the dissimilarity between the station statuses. The ANOSIM R statistic assesses the average ranked dissimilarities in species community composition between different station statuses against those within the same station status. A value of "R" approaching 1.0 signifies notable dissimilarity between the groups, while a value near 0 suggests a balanced distribution of ranks, both high and low, within and across the groups. Additionally, a Multipattern analysis was used to investigate which species are significantly contributing to the difference (R package vegan^52^, R package indicspecies^53^). To visualize the community differences a Non-metric Multidimensional Scaling (NMDS) was used. We calculated an NMDS stress value to determine the reliability of the ordination, with values of > 0.2 indicating a poor fit (R package vegan^52^).

### CRF communities: coral head surveys

To analyse if the abundance, species richness, and diversity of CRF between the coral heads of an active cleaning station and no cleaning station differed for the coral head only analysis, generalized linear mixed models were fitted using the *R* package *glmmTMB*^51^. Treatment and HAS were set as fixed variable, station and depth as random factors. Model assumptions were checked visually with residual plots. Variables were graphically inspected for extreme values.

## Supplementary Information

Below is the link to the electronic supplementary material.

Supplementary Material 1

Supplementary Material 2

Supplementary Material 3

Supplementary Material 4

Supplementary Material 5

Supplementary Material 6

Supplementary Material 7

Supplementary Material 8

Supplementary Material 9

Supplementary Material 10

## Data Availability

The datasets generated and analysed during the current study are available in the figshare repository [https://figshare.com/s/d377c0e724d97dced590].
