# Supplementary material for: Marine cleaning stations as hotspots for cryptobenthic reef fish
Source: Sci Rep. 2026 Apr 2;16:11120. doi: 10.1038/s41598-026-44074-7 (PMC13046782; doi:10.1038/s41598-026-44074-7)
Supplement: Supplementary file 10 — Supplementary Material 10 [file 41598_2026_44074_MOESM10_ESM.pdf]

Supplements

Table S1: A) GLMM ouput for models comparing cryptobenhtic reef fish diversity metrics between benthos around station types (active cleaning station vs no cleaning station). B) GLMM ouput for models comparing cryptobenhtic reef fish diversity metrics between Coral Heads (active cleaning station vs no cleaning station).

A)	**Abundance**				**Shannon Diversity**				**Species Richness**			
Predictors	*Estimates*	*std. Error*	*z-value*	*p*	*Estimates*	*std. Error*	*z-value*	*p*	*Estimates*	*std. Error*	*z-value*	*p*
Intercept	1.06	0.30	3.58	**<0.001**	0.43	0.18	2.41	**0.016**	0.53	0.17	3.04	**0.002**
Treatment	0.85	0.20	4.25	**<0.001**	0.31	0.12	2.58	**0.010**	0.38	0.12	3.23	**0.001**
HAS	0.24	0.10	2.47	**0.014**	0.22	0.06	3.85	**<0.001**	0.20	0.06	3.50	**<0.001**
N	42_{Station}				42_{Station}				42_{Station}			
	29_{Depth}				29_{Depth}				29_{Depth}			

B)	**Abundance**				**Shannon Diversity**				**Species Richness**			
Predictors	*Estimates*	*std. Error*	*z-value*	*p*	*Estimates*	*std. Error*	*z-value*	*p*	*Estimates*	*std. Error*	*z-value*	*p*
Intercept	0.65	0.89	0.73	0.465	0.20	0.32	0.63	0.527	0.74	0.31	2.36	**0.018**
Treatment	1.13	0.35	3.20	**0.001**	0.58	0.13	4.43	**<0.001**	0.70	0.13	5.24	**<0.001**
HAS	0.18	0.27	0.65	0.515	0.02	0.09	0.18	0.858	0.05	0.09	0.57	0.567
N	26_{Station}				26_{Station}				26_{Station}			
	19_{Depth}				19_{Depth}				19_{Depth}			

Table S2: Habitat assessment score (HAS) sheet. Taken from Gratwicke & Speight (2005). Printed to the back of an underwater slate, at each station the HAS score for each category was assessed visually.

	HAS SCORE				
	1	2	3	4	5
**Rugosity** (visual topographic estimate of the substratum in each quadrat)			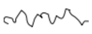	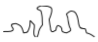	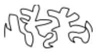
**Variety of growth forms** (stalked/lobed/filamentous/ribbon-like/massive/branching/cylindrical/tube/fan/plate/pinnate/encrusting/other)	<2	3 & 4	5 & 6	7 & 8	9–10
**Height** (visual estimate of average height of habitat architecture) (cm)	0–9	10–19	20–39	40–79	>80
**Refuge size categories** (holes or gaps in habitat architecture or substratum in the following size categories: 1–5, 6–15, 16–30, 31–50 and >50cm)	0–1	2	3	4	5
**Live cover** (total per cent cover of *e.g.* living corals, mangrove roots, seagrass, macroalgae and sponges)	0–19	20–39	40–59	60–79	80–100
**Hard substratum** (%)	0–19	20–39	40–59	60–79	80–100

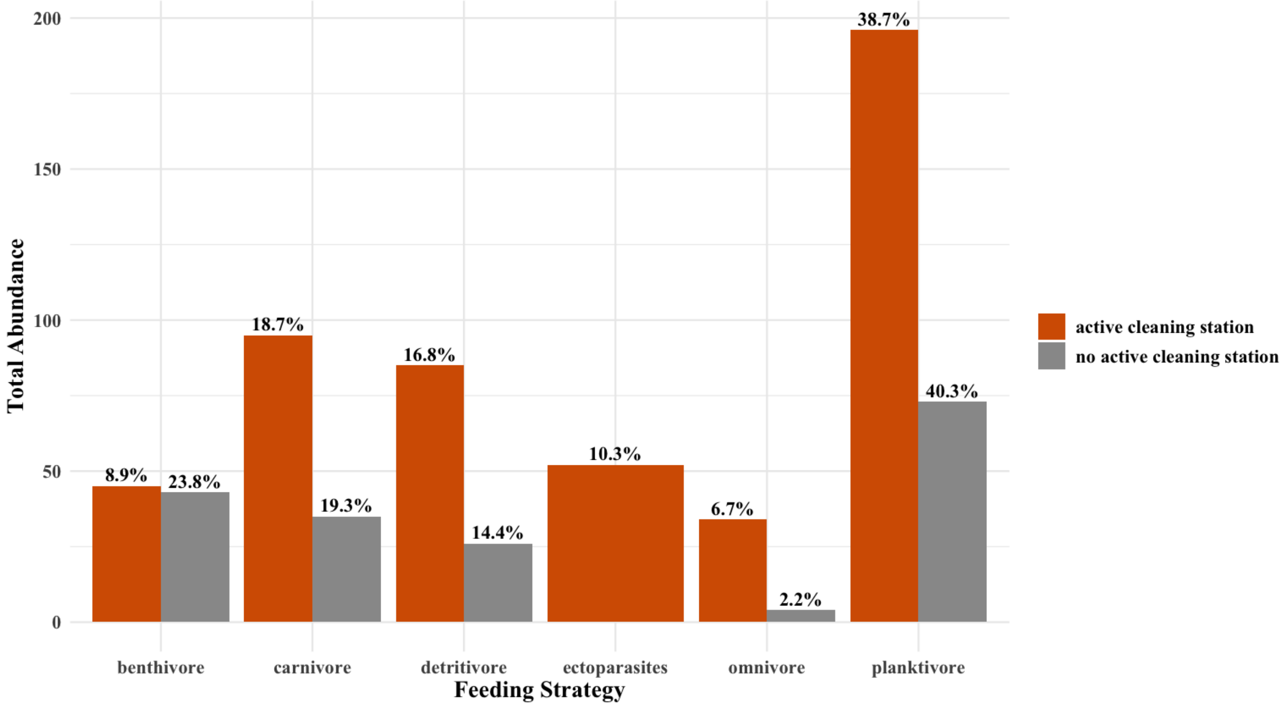

Figure S1 Feeding strategies of CRF found around active vs. non active cleaning stations. *Rough assignment due to the lack of robust information of observed species to primary food sources.All fish might additionally feed on other food sources. Only the cleaner fish (E.lobeli) feeds on Ectoparasites, therefore the feeding strategy is only found around cleaning stations. Percentages added are respectively for the treatments (active/ no cleaning station).*

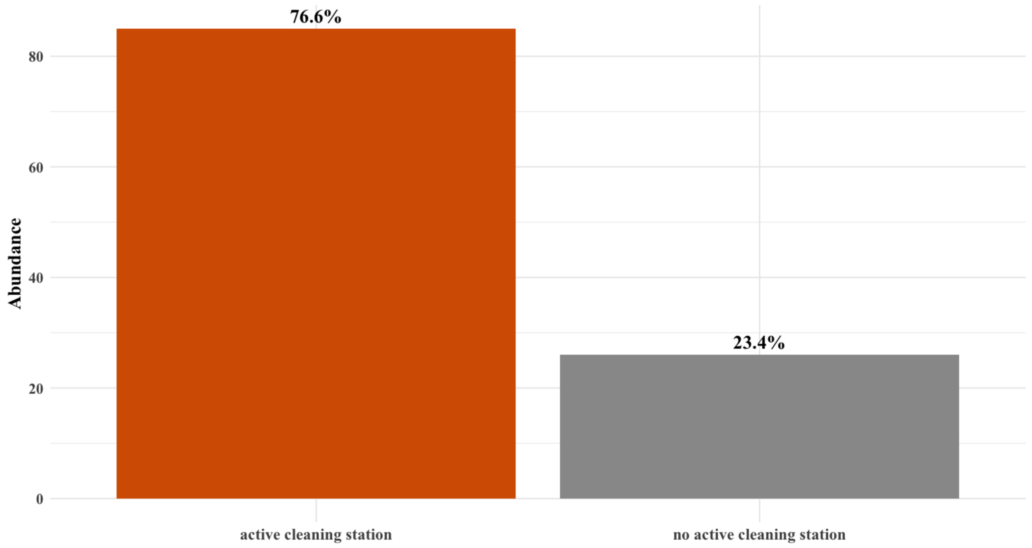

Figure S2 Abundance of detrivores around active vs. no cleaning stations. *Highlights the increase of detrivorous feeders of CRF around active cleaning stations.*

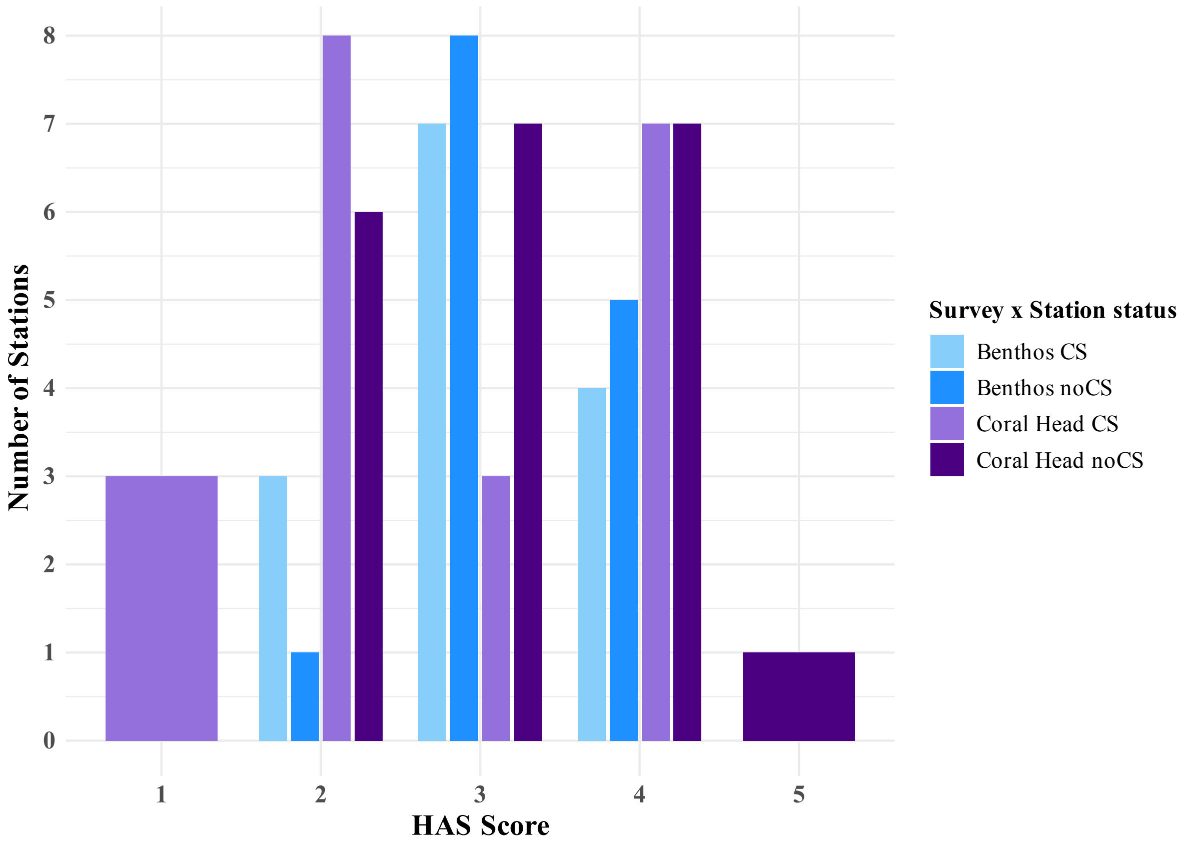

Figure S3 Distribution of HAS Scores by Survey method and Station status *Scores have been derived from categories found in Table 2 by Gratwicke & Speight (2005).*
